# *Plasmodium vivax* in Sub-Saharan Africa: An Advancing Threat to Malaria Elimination?

**DOI:** 10.4269/ajtmh.23-0523

**Published:** 2023-08-28

**Authors:** Mary Aigbiremo Oboh-Imafidon, Peter A. Zimmerman

**Affiliations:** ^1^ Postdoctoral Research Fellow I, Malaria Population Biology, Disease Control and Elimination Theme, Medical Research Council, The Gambia Unit at London School of Hygiene and Tropical Medicine, Serrekunda, Gambia;; ^2^ Professor of International Health, Genetics and Biology, The Center for Global Health & Diseases, Case Western Reserve University, Cleveland, Ohio

Malaria in sub-Saharan Africa (sSA) is principally due to *Plasmodium falciparum.*^1^ The association between *P. falciparum* and severe morbidity and mortality and its capacity to evolve mechanisms to resist and tolerate historically important drugs (sulfadoxine-pyrimethamine, chloroquine,^2^ and artemisinins^3^), requires focus on this dangerous malaria species. Although there are cases of single and mixed species infections of *P. malariae* and *P. ovale *with and without *P. falciparum*,^4^^–^^8^ cases of *P. vivax* have been thought to be absent in sSA due to the erythrocyte silent Duffy-negative phenotype across a substantial number of African ethnicities.^9^^,^^10^ As has become a paradigm in the malaria literature, *P. vivax* appears to require the Duffy antigen to invade human erythrocytes.^11^ A mutation in the GATA-1 transcription factor binding site of the gene promoter (T->C at nucleotide -33) significantly blocks expression of the Duffy antigen on circulating erythrocytes^12^ to establish a protective barrier that strongly inhibits *P. vivax* erythrocyte (but not hepatocyte) invasion.

Over the past 15 years, consistent evidence has accumulated to revise perspectives on *P. vivax* infections and associated illness in African populations.^13^^–^^19^ Much of the evidence emerges when malaria epidemiological studies perform PCR surveys that include detection of *P. vivax*. In this issue of the AJTMH, van Loon et al. add to evidence demonstrating *P. vivax* infections in African populations, this time from the Huye District of Rwanda’s Southern Province.^20^ In their study, they detected *P. vivax* in 21 individuals, all heterozygous carriers of the Duffy-negative SNP (rs2814778 T/C; Duffy-negative allele frequency 1.5%), in the presence of mono and mixed *Plasmodium* species infections. The authors provide evidence that uncomplicated malaria due to *P. vivax* is not rare in southern Rwanda (their PCR-based surveys detected *P. vivax* in consecutive surveys in 8.2% and 7.9% of malaria patients in 2018 and 2019, respectively), and they call attention to the fact that* P. vivax* is rarely studied in Rwanda.

Numerous factors beyond Duffy-negative status contribute to the potential for under-estimating *P. vivax* in sSA. The parasite’s affinity for reticulocytes has been used to explain consistently low density blood stage parasitemia.^21^ With lower parasitemia, there is an increased chance of misdiagnosis, especially by inexperienced microscopists who have not had sufficient in-depth training to identify *P. vivax*. This is particularly true in mixed species infections, with which expert microscopists also struggle to identify low density species.^22^ Routine microscopy training delivered in sSA has under-emphasized the identification of *P. vivax* in blood samples due to the paradigm of the absence of *P. vivax* in Duffy-negative Africans. This continues today, when microscopy training under-exposes trainees to slides including* P. vivax* (in two recent studies <5%^23^ and <10%^24^ of slides contained *P. vivax*) or excludes *P. vivax* slides in training altogether.^25^ Moreover, from 2017 to 2021, of 1.57 billion malaria rapid diagnostic kits (RDTs) purchased for use by sSA national malaria control programs, 79.4% were *P. falciparum*-only tests focused on identifying *P. falciparum* (*Pf* histidine rich-protein II tests) with the remainder being combination tests (Pf/Pan tests) lacking *P. vivax*-specificity.^1^ Thus, the predominant approach for malaria diagnosis across Africa has no ability to specifically detect *P. vivax.*^1^

As the evidence continues to build that *P. vivax* is transmitted throughout much of Duffy-negative Africa, a pertinent question remains to be answered. Is *P. vivax* a threat to malaria elimination in Africa? The answer is complex and dependent on the financial resources available for malaria elimination. Vigilance must remain in studying the therapeutic efficacy of ACTs against *P. falciparum* as the species develops resistance-associated mutations in *Pfkelch13* and other markers that reduce the effectiveness of these essential treatments in Africa.^26^^-^^28^ Therefore, pushing to address *P. vivax* in Africa at the expense of bolstering defenses against *P. falciparum* would foolishly destabilize malaria elimination. However, the evidence that vivax malaria was annually reported from 2010 to 2021 in only 2 of 45 countries in the WHO African Region (Eritrea and Ethiopia) while being detected in at least 29 countries^19^^,^^29^^,^^30^ suggests neglect, potentially facilitating the hidden transmission of vivax malaria and threatening malaria elimination.

Much remains poorly understood regarding *P. vivax* malaria throughout Africa, including the extent of the hypnozoite reservoir, its biomass distribution in infected individuals, and its mechanisms of pathogenesis.^29^^,^^31^ How might we start to turn the page on our myopic view of this very important malaria parasite? Emphasis to microscopists of all malaria species transmitted in Africa, including in-depth training, is essential. The power of RDTs is only partially being realized—new inexpensive RDTs must be better at diagnosing *Plasmodium* species diversity and identifying *P. vivax* specifically. A system must be developed to report species diversity to the WHO because malaria is not a single-species disease. If these initial steps are taken, test-and-treat approaches will need to incorporate reliable and inexpensive point-of-care tests to diagnose glucose-6-phosphate dehydrogenase deficiency to enable safe treatment with primaquine or tafenoquine. It is important to recognize that there are costs with all of these suggested changes, some more than others. However, confronting *P. vivax* wherever it exists in Africa is critical, as its transmission is certain.

As a result of the new paper by van Loon et al.,^20^ we are reminded that *P. vivax* is not only an interesting component of molecular diagnostic surveys: it is also causing malarial illness. Given the overall disregard of *P. vivax*, there is minimal understanding of its impact on individual and public health. So, while attention to falciparum malaria remains essential, failing to study and take action against vivax malaria levies a significant cost against affected populations. It is time to start studying *P. vivax* in Africa. We cannot afford to neglect this species any longer.
